# Platelet signaling in immune landscape: comprehensive mechanism and clinical therapy

**DOI:** 10.1186/s40364-024-00700-y

**Published:** 2024-12-31

**Authors:** Mengyao Yan, Zhe Wang, Zhiwei Qiu, Yimin Cui, Qian Xiang

**Affiliations:** 1 Institute of Clinical Pharmacology, Peking University First Hospital, Beijing, China; 2 Department of Pharmacy Administration and Clinical Pharmacy, School of Pharmaceutical Sciences, Peking University Health Science Center, Beijing, China

**Keywords:** Platelet, Inflammation, Immunity, Clinical strategies

## Abstract

Platelets are essential for blood clotting and maintaining normal hemostasis. In pathological conditions, platelets are increasingly recognized as crucial regulatory factors in various immune-mediated inflammatory diseases. Resting platelets are induced by various factors such as immune complexes through Fc receptors, platelet-targeting autoantibodies and other platelet-activating stimuli. Platelet activation in immunological processes involves the release of immune activation stimuli, antigen presentation and interaction with immune cells. Platelets participate in both the innate immune system (neutrophils, monocytes/macrophages, dendritic cells (DCs) and Natural Killer (NK) cells and the adaptive immune system (T and B cells). Clinical therapeutic strategies include targeting platelet activation, platelet-immune cell interaction and platelet-endothelial cell interaction, which display positive development prospects. Understanding the mechanisms of platelets in immunity is important, and developing targeted modulations of these mechanisms will pave the way for promising therapeutic strategies.

## Graphical abstract

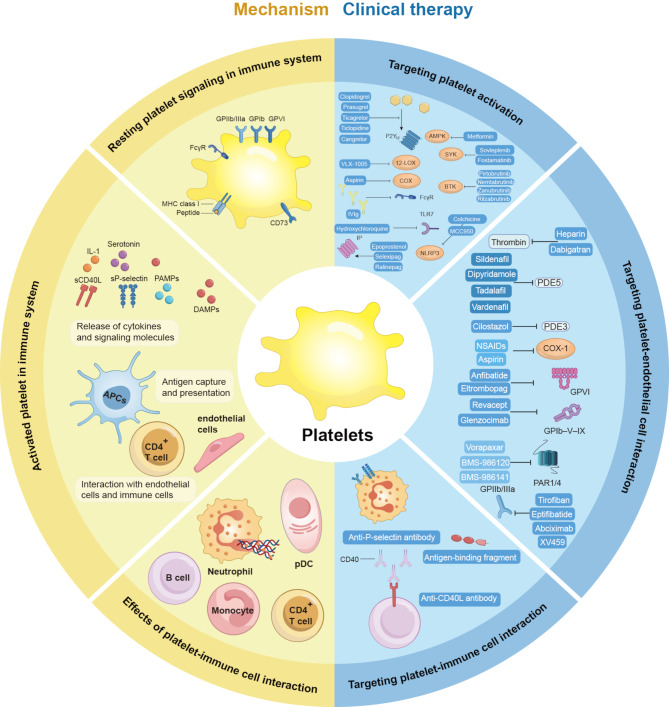

## Introduction

Platelets, a non-nucleated blood component, are the main cell type engaged in hemostasis and thrombosis regulation [1]. Platelets have a short lifespan, typically 4–6 days in mice and 5–9 days in humans and are cleared in the spleen and liver [2]. Millions of platelets must be continuously produced every hour to maintain their physiological blood counts and prevent the risk of bleeding [3]. The production of platelets by megakaryocytes is a systematic process that commonly occurs in the bone marrow and each platelet produces 1000–3000 platelets after multiple divisions [4]. Platelets play a major role in the process of primary hemostasis and thrombosis and their main function is to prevent bleeding after vascular injury by quickly binding with damaged blood vessels and forming blood clots [5].

In addition to their significance in hemostasis and thrombosis, platelets serve as mediators of inflammation and immune response directly linking thrombotic diseases to inflammatory processes [6]. Firstly, platelets express numerous receptors and store hundreds of secretory products that play a crucial role functional response [3]. All of these components offer potential new pathways for drug targeting to address various immune-mediated inflammatory diseases [7]. For example, platelet factor 4 (PF4) has a novel function. It is released from platelet α-granules upon activation and has the ability to form immune complexes, activating platelets and neutrophils through Fc receptors [8]. Secondly, platelets secrete proinflammatory cytokines, chemokines and biological response modifiers, such as the CD40 ligand, as well as lipid metabolites that can function as autocrine and/or paracrine mediators [9].

Platelets are potential key drivers of the inflammatory response and are crucial for immune reactions [10]. This review aims to provide an overview of recent research indicating that platelets may become activated in various immune-mediated inflammatory diseases, change their phenotype to express surface glycoproteins and produce soluble substances that affect immune cell activity [11]. Activated platelets play a significant role in immune dysregulation in multiple immune-mediated inflammatory diseases by influencing the phenotype of both innate and adaptive immune cells [12]. Additionally, by amplifying local inflammation and fibrosis, activated platelets promote end-organ damage and contribute to the development of several immune-mediated inflammatory diseases due to their enhanced aggregation [13]. Research linking platelet to the development of inflammation and immune diseases is emerging, and more and more therapeutic strategies for platelet activation or platelet-derived factors are being developed and tested in clinical studies.

## The function of platelets in various diseases

**Fig. 1:**
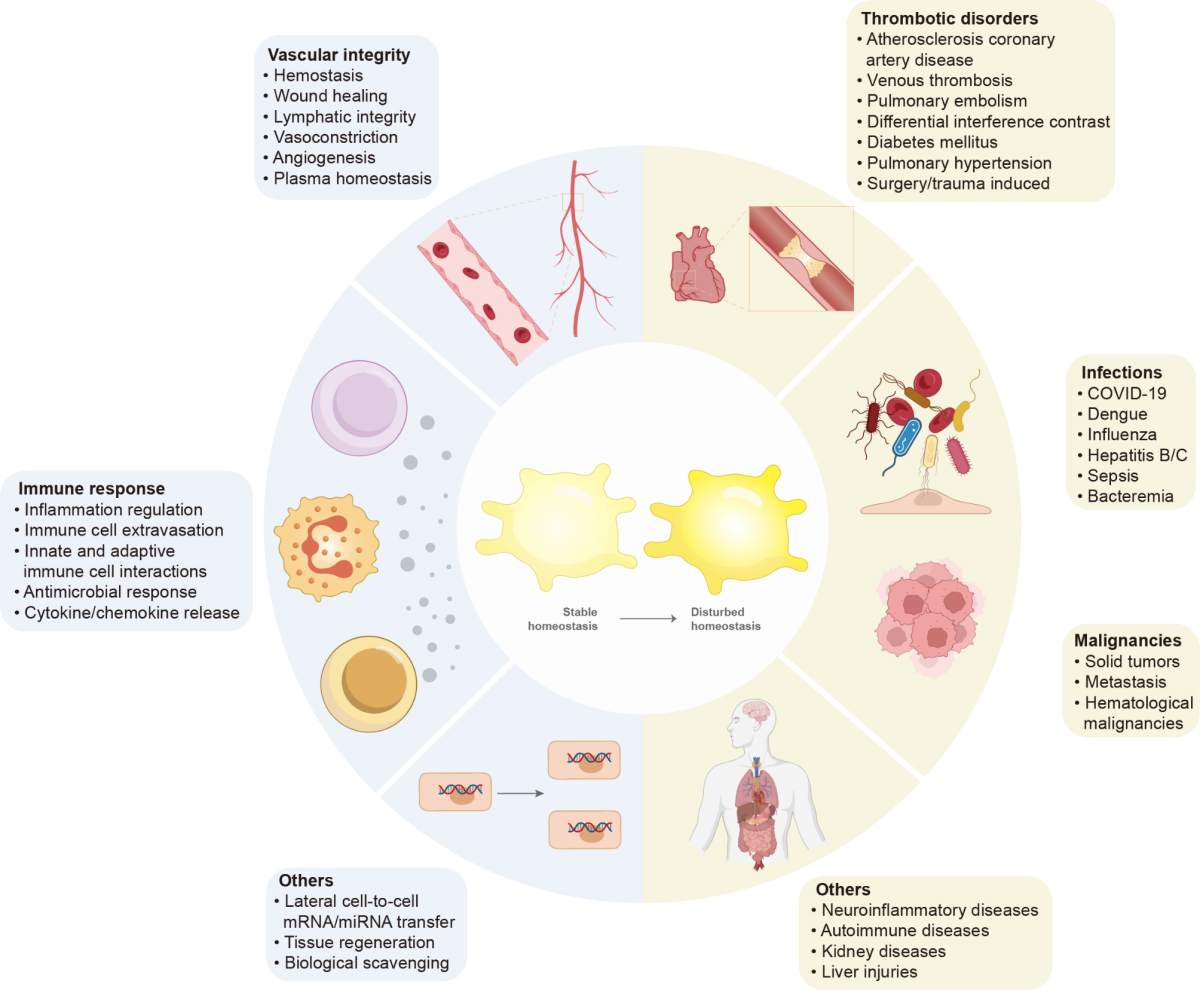
Illustration of platelets in different conditions and diseases. In stable homeostasis, platelets perform a multitude of physiological functions by preserving vascular integrity, maintaining the balance of the immune system, and other conditions. Platelets preserve vascular integrity by influencing blood hemostasis, wound healing, lymphatic integrity, vasoconstriction, angiogenesis and plasma homeostasis. Platelets take part in the immune response by regulating inflammatory reactions, influencing immune cell extravasation, interacting with innate and adaptive immune cells, regulating antimicrobial responses and controlling cytokine/chemokine release. Besides, platelets also perform other physiological functions such as absorbing or transmitting mRNA or miRNA to other cells. In disturbed homeostasis, platelets affect the pathogenesis of several disease states, mainly including thrombotic disorders, malignancies, infections and other related diseases. Platelets affect thrombotic disorders mainly including atherosclerosis coronary artery disease, venous thrombosis, pulmonary embolism, differential interference contras, diabetes mellitus, pulmonary hypertension, surgery/trauma-induced thrombotic disorders. Platelets are intricately linked to infections and malignancies, including COVID-19, dengue, influenza, hepatitis B/C, sepsis, bacteremia, solid tumors, metastasis and hematological malignancies. Platelets also affect neuroinflammatory diseases, autoimmune diseases, kidney diseases and liver injuries

In healthy conditions, platelets are essential for thrombosis and hemostasis. Recent studies have revealed that platelets have additional physiological functions, including maintaining vascular integrity [14], immuno-regulation [15] and tissue regeneration [16] (Fig. 1). Platelets maintain vascular integrity by affecting blood hemostasis, wound healing, lymphatic integrity, vasoconstriction, angiogenesis and plasma homeostasis. For example, platelets exert their hemostatic function by promoting the recruitment of leukocytes to the inflammatory site and subsequently maintaining vascular integrity to prevent bleeding at the site of leukocyte infiltration [17]. Furthermore, in the process of hemostasis and inflammation, platelet activation leads to the activation of its related receptors and the production of related secretions, as well as the interaction between platelets and neutrophils and monocytes/macrophages, which store and release inflammatory cytokines [18, 19]. Additionally, platelet al.so enter some tissues like the liver and brain, promotes both platelet adhesion, inflammatory actions, and increasing the frequency of platelet-immune cell interactions [20, 21]. The physiological functions of platelets are mediated by mRNA or miRNA transfer to or reception from other cells. Platelets release active metabolites such as thromboxane A_2_, adenosine diphosphate (ADP), serotonin as well as proteins such as α-granule proteins, which play a role in inflammation and tissue regeneration [22].

In pathological conditions, platelets are gradually becoming important regulatory factors, and their significance in the occurrence and progression of cardiovascular disease has been extensively proven [23]. Platelets have also been demonstrated to play a crucial in the pathophysiology of thrombotic disorders [24], cancer [25], infections such as severe acute respiratory syndrome coronavirus-2 (SARS-CoV-2) leading to coronavirus disease 2019 (COVID-19) [26] and other related diseases (Fig. 1).

## Mechanism of platelets in immune system

**Fig. 2:**
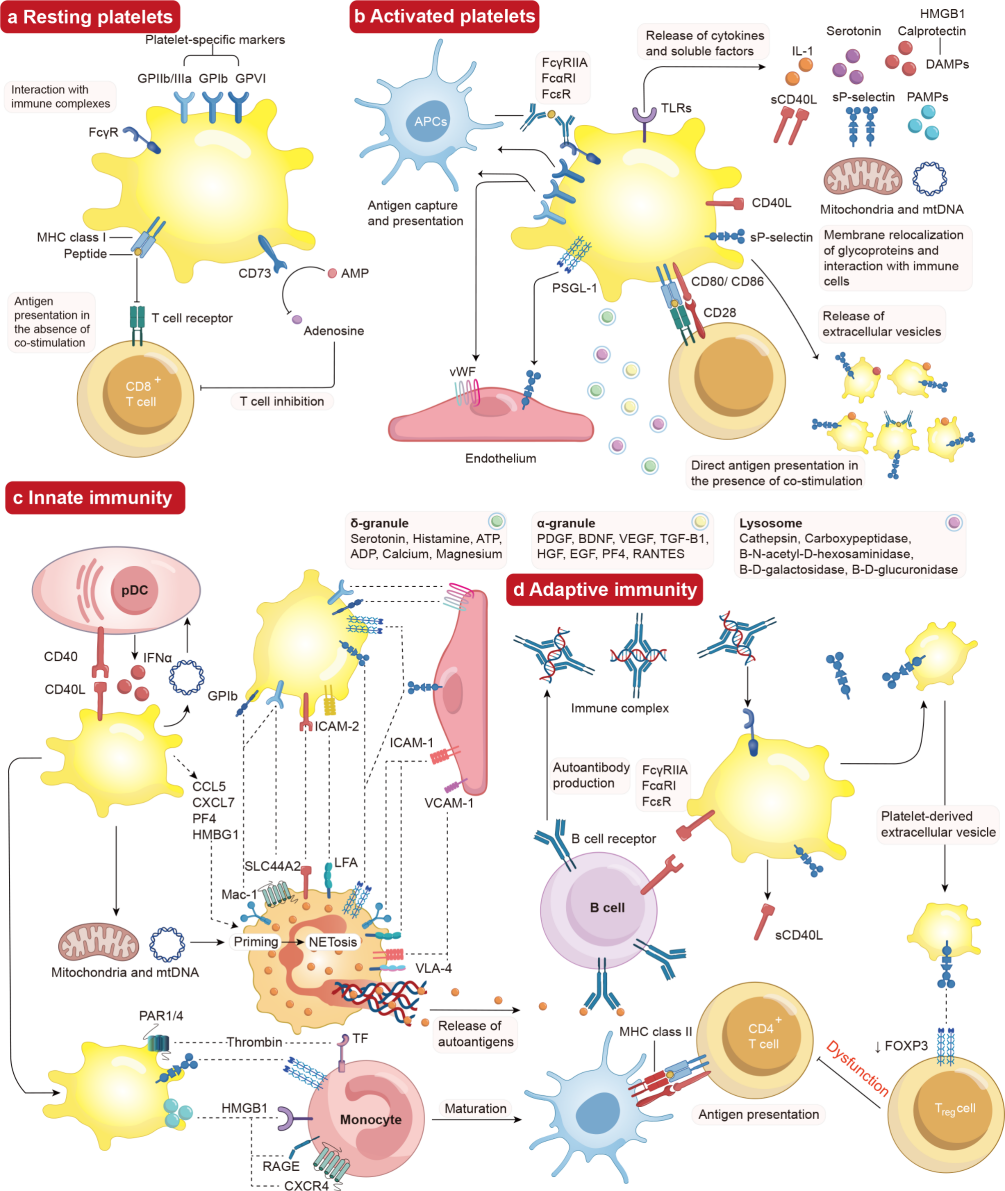
Effects of interaction between activated platelets and the immune system. (**a**) Resting Platelet-specific receptors, including the glycoproteins GPIIb/IIIa, GPIb, GPVI, CD73, MHC class I and FcγR are expressed by resting platelets. Resting platelets express MHC Class I in the lack of costimulatory compounds and express ectonucleosidase CD73, which inhibits CD8^+^ T cells that convert AMP into the anti-inflammatory properties’ adenosine. Resting platelets also express FcγR, which assists in removing circulating immune complexes. (**b**) Activated platelets produce DAMPs such as calprotectin and HMGB1, molecules like sP-selectin and sCD40L, as well as serotonin and cytokines like IL-1. Additionally, activated platelets cause the extrusion of mitochondria and mtDNA towards the extracellular environment. APCs grab platelet antigens, which then remove immune complexes for processing and presentation to the immune system. Besides, platelets also express MHC class I and co-stimulatory molecules CD86/CD80. They directly present antigens to CD8^+^ T cells, promoting their activation. Platelet activation reset surface glycoproteins, such as P-selectin and CD40L, aiding in intercellular communication with immune cells. Activated platelets also promote the release of platelet-derived extracellular vesicles that included granules, lysosomes, CD40L, HMGB1, P-selectin and IL-1. Under endothelial cell activation, activated platelet surface receptors like GPIIb/IIIa and PSGL-1 binds to vWF and P-selectin on the endothelium, facilitating communication with immune cells. (**c**) Innate immunity is stimulated by platelets through interactions between CD40L and CD40, leading to the assembly of platelets with pDCs and boosting the production of IFNα in reaction to bloodstream immune complexes. Platelets communicate with monocytes and neutrophils via various surface receptors, promoting the maturation of monocytes into APCs and stimulating neutrophil activity. Platelets generate mitochondria and mtDNA, which activate neutrophils and cause the creation of NETs. Autoantigens are liberated, dealt with by APCs, and then delivered to lymphocytes. (**d**) Adaptive immunity is triggered by platelets, which express membrane CD40L and sCD40L, eliciting B cell reactions and the generation of autoantibodies. T_reg_ cells interact with P-selectin-positive platelets and extracellular vesicles-derived platelets, resulting in the downregulation of transcription factor FOXP3 and affecting T_reg_ cell function

Platelets in the bloodstream are typically in a resting state (Fig. 2a) and become activated in response to stimuli like bacterial infections or vascular wall. Upon activation, platelets undergo a complex process involving adhesion and signaling molecules (Fig. 2a), particularly in platelet remodeling [27]. This transformation prompts platelets to shift from a discoid shape to a more spherical form, especially adopting a spherical conformation with lamellipodia, enhancing their ability to interact with other cells. This remodeling is triggered by the release of intracellular calcium, activating the actin-myosin cytoskeleton and increasing the average platelet volume [28]. For example, For instance, patients with conditions like active systemic lupus erythematosus (SLE) [29], inflammatory bowel diseases [30] and rheumatoid arthritis (RA) [31] often exhibit higher platelet volumes. Additionally, activated platelets expose negatively charged phospholipids on their surface, facilitating the binding and activation of coagulation factors, tissue factors, and thrombus formation [32]. Activated platelets release delta-granules, alpha-granules and lysosomes, further amplifying platelet activation through a positive feedback loop mediated by ADP or adenosine triphosphate (ATP) complement [33]. Glycoproteins like P-selectin and CD40 ligand (CD40L) relocate from cytoplasmic granules to the active platelet surface, promoting interaction with immune cells [34]. The generation of platelet-derived extracellular vesicles containing various molecules in activated platelets allows the diffusion of platelet components to fluids and tissues that platelets typically cannot access, such as the lymphatic and central nervous systems, as observed in inflammatory arthritis and neuroinflammation [35, 36]. In summary, activated platelets alter their phenotype and morphology, potentially enhancing their interaction with immune cells.

### Resting platelet in immune system

Resting platelets express many platelet-specific markers on the surface including Fcγ receptor, MHC class I, CD73, and the glycoproteins GPIIb/IIIa, GPIb and GPVI. This enables them to participate in the circulating immune system. Resting platelets exhibit morphological changes, adhesion, aggregation and release reactions under the influence of many factors, which mainly include immune complexes through Fc receptors [37], platelet-targeting autoantibodies [38, 39] and platelet-activating factors such as toll-like receptors (TLRs) [40], NOD-like receptor family pyrin domain containing 3 (NLRP3) inflammasome pathway [41], collagen [42, 43] and Raynaud’s phenomenon [44]. These factors encourage platelets to transition from a resting state to an active state in the circulating immune system.

#### Immune complexes through fc receptors

Fc receptors are reflected in the variety of molecules that bind antibodies and immune complexes, mainly consisting of Fcγ receptor IIA (FcγRIIA), Fcα receptor I (FcαRI) and Fcε receptor (FcεR) [45]. Human platelets, neutrophils, monocytes, macrophages, certain dendritic cells, and other granulocyte subpopulations express FcγRIIA, also known to as CD32 [46]. FcγRIIA’s unique property is its affinity for IgG immune complexes, which specifically bind to antigens when they are contained within the Fc domain [47]. However, FcγRIIA exhibits less affinity for monomeric IgG [48]. Platelets are the most abundant FcγRIIA pool in the blood because they express FcγRIIA more frequently than other blood cells do. FcγRIIA functions as a whole membrane receptor on the surface of platelets and is most frequently found in homodimers. The cytoplasmic tail of FcγRIIA has a tyrosine-based immune receptor activation motif, which is an essential component for signal activity [49]. Besides, efgartigimod is a human IgG1 antibody Fc-fragment, a natural ligand of the neonatal Fc receptor, and it induces a rapid reduction of total IgG levels, which is associated with clinically relevant increases in platelet counts in ITP [50].

When activated, platelets expressing FcγRIIA generate platelet-derived extracellular vesicles that engage in interactions with circulating neutrophils and contribute to the production of thrombi in some organs [51]. Furthermore, FcγRIIA-stimulated platelets release mitochondria, facilitating their dissociation or embedding into extracellular vesicles formed from platelets. These mitochondria act as autoantigens and damage-associated molecular patterns (DAMPs) that trigger autoimmune reactions [46]. The platelet activation mechanism may be applicable to a wide range of immune-mediated inflammatory diseases, as immune complexes containing IgG have been found in diseases, such as sepsis, ischemia-reperfusion (IR) injury, RA, systemic sclerosis, vasculitis and Sjögren’s syndromes [52].

Platelets also express FcαRI, commonly referred to as CD89, acting as a regulator between anti-proinflammatory responses of IgA [53]. FcεR referred to as CD23, is also expressed on platelets and functions as a modulator between anti-proinflammatory responses of IgE. Due to immune complexes rich in IgA or IgE in immune-mediated inflammatory diseases, such as sepsis, IR injury, RA, systemic sclerosis, vasculitis and Sjögren’s syndromes [54, 55]. Under platelet activation, these data highlight that the additional pathways of Fc and the significance of immune complex Fc receptor interactions in immune-mediated inflammatory diseases.

#### Platelet-targeting autoantibodies

Platelet-targeting autoantibodies include antiphospholipid antibodies and antiplatelet antibodies. Antiphospholipid antibodies are a characteristic feature of antiphospholipid syndrome (APS) and are seen in systemic autoimmune diseases, mainly including SLE [56], systemic sclerosis [57], Sjögren’s syndrome [58] and other immune-mediated inflammatory diseases [59]. These antibodies can directly bind to platelets through phospholipids found in the cell membrane or through the platelet glycoprotein (GP) Ibα component of GPIb, also known as CD42, which forms part of the von Willebrand factor (vWF) receptor and stimulates platelet activation and aggregate formation [60]. Thus, blocking or depleting any of these components is known to exacerbate bleeding after traumatic vascular damage [61]. Crucially, these antiphospholipid antibodies also bind to the endolysosomal phospholipid lysobisphosphatidic acid provided by cell surface-expressed endothelial protein C receptor (EPCR) on monocytes and dendritic cells (DCs). This enhances the signal transduction between downstream interferons activated by TLR7 through immune complexes and complement-mediated activation of tissue factor (TF), thus linking the proinflammatory signaling and thrombosis [62]. Additionally, antiphospholipid antibodies increase substances that activate platelets in the blood, such as soluble CD40L and chemokines produced by platelets [63].

An established pathophysiological mechanism of immune thrombocytopenia (ITP) pathogenesis includes the recognition of membrane glycoprotein complexes, mainly GPIb/IX and GPIIb/IIIa, by anti-platelet and anti-megakaryocyte antibodies [64]. Antiplatelet antibodies can also directly bind to the surface of platelets and activate the related complement, leading to platelet activation as well as immune-mediated disruption via cytotoxicity or antibody-dependent phagocytosis. Membrane glycoprotein complexes GPIb-IX, including as GPIbα, GPIbβ, GPIX, and GPIa, are recognized by anti-megakaryocyte antibodies in combination with the GPIIb/IIIa complex, commonly referred to as CD41/CD61, which is present in ITP patients [65].

#### Other platelet-activating factors

##### TLRs

Platelets express TLRs, which play a role in activating this receptor and regulating innate immunity in the bloodstream [66]. In response to viral reactivation or tissue injury, there is an increase in molecular patterns associated with various infections and circulating levels of DAMPs, which can trigger platelet TLRs [67].

TLR4 and TLR2 are the primary TLRs involved in platelet activation and aggregation when exposed to lipopolysaccharide (LPS). Study on mice lacking TLR2 or TLR4 shows a decrease in platelet count compared to wild-type mice, indicating that these TLRs enhance the anti-infective function of platelets [68]. High mobility group box 1 (HMGB1) can potentially activate platelets through TLR2 and TLR4 [69]. Importantly, the activation of TLR2, particularly results in ROS production and uptake of molecules like fibrinogen. In contrast, activation of TLR4 mediates the formation of platelet–neutrophil complexes. Besides, stimulation of TLR4 results in decreased Akt/protein kinase B phosphorylation, conditioned by enhanced protein phosphatase 2 A activity. TLR4-mediated signaling induces platelet adhesion and facilitates ristocetin-induced platelet agglutination, which aggravates thrombosis and autoimmune diseases, such as RA, SLE, systemic sclerosis, Sjogren’s syndrome, psoriasis, multiple sclerosis, and autoimmune diabetes [70].

Platelets express TLR3 both on surface and in cytoplasm and that these cells response to the synthetic analog of dsRNA (poly I: C) by increasing surface expression of TLR4 and CD62P, and releasing CXCL4 and IL-1β [71]. Besides, TLR3 activation resulted in reduced platelet production in vitro and interferon-β release through the PI3K-Akt and NF-κB signaling pathways. TLR3 ligands potentiated the aggregation mediated by classic platelet agonists [72].

The endosomal TLR7 is expressed constitutively in platelets. TLR7 activates the Myd88-dependent signaling cascade, eliciting an immune response. Dysregulation and variations in TLR7 expression are implicated in several autoimmune disorders like primary Sjogren’s syndrome-related thrombocytopenia. In genetically predisposed individuals, factors such as infections, endocrinological abnormality and metabolic abnormalities can cause TLR7 dysregulation, aggravating primary Sjogren’s syndrome symptoms and progression [73]. TLR7 agonists also activate platelets and increase the levels of platelet-neutrophil aggregates in the bloodstream, and this effect depends on P-selectin binding to P-selectin glycoprotein ligand 1 (PSGL1), which relieve disease progression of SLE [74]. During infection with encephalomyocarditis virus, platelet-TLR7 stimulation mediates formation of large platelet-neutrophil aggregates, both in mouse and human blood. Intriguingly, this process results in internalization of platelet CD41-fragments by neutrophils with no influence on platelet prothrombotic properties. The mechanism includes TLR7-mediated platelet granule release, translocation of P-selectin to the cell surface, and a consequent increase in platelet-neutrophil adhesion. Viral infection of platelet-depleted mice also leads to increased mortality. Transfusion of wild-type, TLR7-expressing platelets into TLR7-deficient mice caused a drop in platelet count and increased survival post encephalomyocarditis virus infection [75]. Moreover, a mice model with this mutation exhibits severe thrombocytopenia, indicating platelet activation. A family with severe and early-onset SLE has been shown to have a gain-of-function mutation in the *TLR7* gene [76].

TLR9 is also expressed in platelets. In reaction to CpG dinucleotides, which are present in pathogen and mitochondrial DNA, it promotes platelet activation and the uptake of oxLDLs from the circulation. TLR9 recognizes viruses and detects endogenous ligands generated under oxidative stress-related pathophysiological conditions. For example, platelet hyperreactivity and thrombosis by TLR9 ligands induces IRAK1 and AKT phosphorylation, and it is Src kinase-dependent in atherosclerosis. Physiological platelet agonists act synergistically with TLR9 ligands by inducing TLR9 expression on the platelet surface [77]. Besides, platelets are activated by DNA viruses, such as Epstein-Barr virus (EBV), through TLR9 following the endocytosis of viral particles in chronic SLE and long COVID infections [78].

##### NLRP3 inflammasome pathway

The canonical NLRP3 inflammasome is a crucial component in the onset and spread of venous thrombosis. It responds to DAMPs and pathogen-associated molecular patterns (PAMPs), undergoes gain-of-function mutations in immune-mediated inflammatory diseases and produces the prototype inflammatory cytokines interleukin (IL)-1β and IL-18 [79]. Platelets express NLRP3, which is involved in their activation and immunological functions. NLRP3 inflammasomes can be triggered by various stimuli, including RNA virus infection through mitochondrial antiviral signaling proteins [80], oxidized mitochondrial DNA, mitochondrial reactive oxygen species (ROS) [81], macrophages activated by neutrophils and neutrophil extracellular traps (NETs) [82], platelet-derived HMGB1 signaling via TLR4 [83], which stimulates platelet activation and the generation of IL-1β and IL-18. For example, activating the NLRP3 inflammasome pathway stimulates the generation of IL-1β and IL-18 from injured cells, enhances vascular permeability, and stimulates the recruitment and transportation of macrophages and neutrophils. Consequently, leukocyte-produced IL-1β and IL-18 can stimulate the endothelium and enhance platelet recruitment, activation, and aggregation [84]. Activation of the NLRP3 inflammasomes contributes to GSDMD-pore formation in platelet pyroptosis. Gasdermin D-dependent platelet pyroptosis is induced by high levels of S100A8/A9 targeting TLR4. Pyroptotic platelet-derived oxidized mitochondrial DNA potentially promotes NET formation, which contributed to platelet pyroptosis by releasing S100A8/A9, forming a positive feedback loop that led to the excessive release of inflammatory cytokines [85].

##### Raynaud’s phenomenon

Raynaud’s phenomenon is characterized by small artery spasms caused by microvascular system disorders, which are observed in all patients with systemic sclerosis, RA and SLE [86]. Under cold or mental stress, the skin of fingers, toes and other parts becomes pale, cyanotic, and flushed, accompanied by pain and abnormal sensations [87]. Localized ischemia-reperfusion injury destroys endothelial cells, exposing collagen and releasing reactive oxygen species (ROS). These molecules combine with collagen, bind to GPVI, and trigger platelet activation [88]. Increased counts of neutrophils, and the role of PSGL-1/P-selectin interaction in the production of NETs and higher circulating levels of platelet-leukocyte aggregates are characteristics of individual with primary Raynaud’s phenomenon [89].

### Activated platelet in immune system

Platelets are activated by various physical, chemical, and biological factors, leading to rapid rearrangement and conformational changes in the granule membrane glycoproteins within platelets. This process enhances adhesion ability and may result in the release of pro-inflammatory factors to contribute to the immune response during hemostasis [90, 91]. Platelet activation leads to the release of immune activation stimuli such as cytokines [92], signaling molecules [93], antigen presentation [94], and interactions with endothelial cells [95] and immune cells [24] (Fig. 1b).

#### Release of cytokines and signaling molecules

Activated platelets secrete a plethora of chemokines including CXCL4 or platelet factor 4 (PF4), CCL5, CXCL12 or stromal cell derived factor-1α (SDF-1α), IL-1β and serotonin, which initiate or promote local inflammatory processes at sites of vascular injury [96, 97].

CXCL4 and CCL5 are platelet derived cytokines highly expressed in patients with atherosclerosis with a proposed atheroprotective role. Recently study demonstrates that these chemokines facilitate the recruitment of immune cells into plaques, creating an inflammatory and atherosclerotic environment [98]. Importantly, these chemokines now serve a more significant role beyond chemotaxis as they can stimulate DCs to produce type I interferon through TLR9-dependent mechanisms, contributing to the development of autoimmunity [99]. Similarly, thrombomodulin, an antimicrobial peptide produced from α-granules post platelet activation, is derived from the carboxyl terminus of platelet chemokines [100].

SDF-1α, also known as CXCL12, have both enhancing and inhibitory effects on T cells, neutrophils and eosinophils as chemotactic agents [101, 102]. In inflammatory environments, the adhesion of monocytes to platelets is a common occurrence that leads to NF-κB translocation to the nucleus, where it triggers monocyte expression of IL-8 [103]. The mobilization of neutrophils to the blood by CXCL8 is directly counteracted by CXCL12, which retains leukocytes in the bone marrow through the cell surface receptor CXCR4. CXCR4 is highly expressed in various malignant cells. Platelets, in turn, are activated by CXCL12 and produce CXCL12, which is essential for monocyte differentiation and neointimal formation [104].

IL-1β is the best described newly synthesized protein after pro-RNA splicing in platelets. This cytokine is linked to endothelial dysfunction and coagulation disorders in many inflammatory, infectious, and cardiovascular diseases. It activates cells of the immune system and of the vascular wall through IL-1 receptor signaling promoting inflammation, angiogenesis, and differentiation of myeloid progenitor cells [105]. For example, LPS activates platelets through TLR4, aiding productive sepsis, with stimulated splicing and translation of stored heteronuclear pro-IL-1β RNA. IL-1β is a platelet agonist, that IL-1β acts through an autocrine stimulatory loop, that an IL-1β autocrine loop is required to amplify platelet activation by LPS, and that platelets immobilized in occlusive thrombi are activated over time to produce IL-1β. IL-1 is a new platelet agonist that promotes its own synthesis, connecting thrombosis with immunity [106].

Platelets are the main source of peripheral serotonin. Serotonin, sometimes referred to as 5-HT, is a neurotransmitter that is synthesized from tryptophan and is taken up by platelets in the blood and stored in dense granules [107]. In a mice model of autoimmune RA, serotonin released from activated platelets stimulates the permeability of the joint vascular system. This may potentially facilitate the entry of platelet-derived extracellular vesicles and other blood-borne molecules into the inflammation joint and lymphatic system [108]. Activated platelets produce serotonin, which may subsequently affect immune cell activity because the majority of immune cells carry one or more types of serotonin receptors [109]. For example, tryptophan 5-hydroxylase 1 (Tph1)^−/−^ mice, deficient in serotonin production, exhibit a reduction in the migration of neutrophils to the inflammatory site upon infection stimulation due to impaired diapause. Furthermore, serotonin activates of immature T cells in mice and promotes the proliferation of CD4^+^ T helper cells in humans via 5-HT_7_ and 5-HT_1B_ receptors [110].

#### Antigen presentation

The major histocompatibility complex (MHC) class I is a diversified group of receptors located on the cell surface found on every nucleated cells in the body, including platelets [111]. MHC class I molecules, referred to as human leukocyte antigens, are essential components of the immune system by distinguishing self from nonself and presenting foreign antigens to other immune cells [112]. MHC class I molecules within cells are rearranged and brought to the surface by activated platelets. These molecules can interact with T cells to create a synapse, present antigens, and promote their activation and proliferation. They can also work in concert with other molecules, such as the co-stimulatory molecule CD86. In an ovalbumin-based antigen-presenting model, platelet-derived extracellular vesicles can improve T cell activation by transferring the mechanism for antigen processing and presentation [113]. Additionally, the high expression of platelet MHC-I promotes antigen-specific cross-presentation and interactions with CD8^+^ T lymphocytes. Notably, while platelet MHC-I expression is significantly increased in humans and mice, platelet MHC-II expression remains unchanged. Megakaryocytes utilize MHC class I to deliver antigens and increase the production of systemically produced LPS and INF-γ, which in turn enhance CD4^+^ T cell activation and proliferation by expressing MHC class II [94].

Extracellular vesicles generated by platelets are decorated with autoantibodies due to the presence of citrullinated autoantigens, that include vimentin and citrullinated fibrinogen, in the synovial fluid of RA individuals [114]. Moreover, active platelets might be phagocytized by antigen-presenting cells, which will treat them as foreign antigens. This mechanism can increase the generation of antiplatelet antibodies, which are present in ITP, SLE and other immune-mediated inflammatory diseases [65].

#### Interaction with endothelial cells and immune cells

The communication between active platelets and endothelial cells limits pathogen penetration and strengthens the local immune response by immune thrombosis. For instance, platelets promote the generation of IL-1β, activate the function of endothelial cells and increase endothelium permeability, leading to tissue regeneration and the local immune response [115, 116]. Additionally, platelet-endothelium interactions include P-selectin binding to endothelial PSGL1 and other glycoproteins like GPIb binding to endothelial vWF. Subsequently, endothelial cells may be stimulated by inflammatory agents through CD154 or its receptor CD40, both of which are expressed on activated platelets. This interaction contributes to the formation of thrombi and the migration of immune cells [34, 117]. Moreover, injecting extracellular vesicles produced from human platelets into immunodeficient mice results in diffuse endothelial damage, at least partially due to the expression of HMGB1 on the vesicle surface, which activates the capacity of neutrophils [118].

The communication between platelet and immune cell receptors plays a crucial role in multiple immune-mediated inflammatory diseases [24]. While resting platelets lack the ability to stimulate the immune system, they can still exert inhibition by expressing MHC class I molecules when co-stimulatory markers such as CD80 and CD86 are absent [119]. Adhesion molecules, which improve interaction with immune cells, can be produced by activated platelets. The most well-characterized is P-selectin, a lectin that relocates to the platelet surface after activation and attaches to PSGL1 [120]. The leukocyte integrin macrophage antigen-1 (Mac-1), also known as αMβ2 integrin, is another important adhesion molecule that plays a role in the interactions between platelets and immune cells. It reacts directly with platelet GPIbα or indirectly with GPIIb/IIIa via leukocyte-fibrinogen aggregation [121]. leukocytes and platelets both express CD40L, a homophilic binding-related element of the signaling activated lymphocyte molecule family. Platelet CD40L also facilitates physical contact and communication with immune cells that express CD40 and CD84 [34]. Interestingly, all TLR1-TLR10 mRNA transcripts are present in platelets. PAMP receptors known as TLRs have an important function in initiating the innate immune response to invading pathogens [122].

The interaction between platelets with neutrophils, monocytes, lymphocytes, leukocytes and Kupffer cells is critical for initiating the immune response in multiple immune-mediated inflammatory diseases [123]. Patients with these conditions exhibit elevated circulation levels of soluble P-selectin and platelet-derived extracellular vesicles in circulation, indicating platelet activation. This activation may be primary factor leading to increased contents of platelet-neutrophils, platelet-leukocytes and platelet-monocytes, aggregates [124]. Additionally, neutrophils aggressively search the bloodstream for P-selectin-positive activated platelets. These platelets predominantly bind to PSGL1 and interact with the neutrophil uropod, resulting in tissue injury. The infusion of wild-type platelets partially restores the damaged vascular adhesion of leukocytes in P-selectin-deficient mice by inhibiting the expression of endothelial P-selectin and platelet-leukocyte aggregation. This suggests that platelets assist in immune cells migration to inflammatory sites to enhance pathogen elimination [125, 126]. The platelet-derived GPIbα plays a role in platelet adhesion and the inflammatory functions of platelets, leading to increased recruitment of leukocytes to the inflamed central nervous system [21]. Recently, patients with non-alcoholic steatohepatitis and subsequent liver cancer cause platelets to interact primarily with Kupffer cells, leading to an increase in intrahepatic platelet numbers [20].

### Effects of platelet-immune cell interaction

**Fig. 3:**
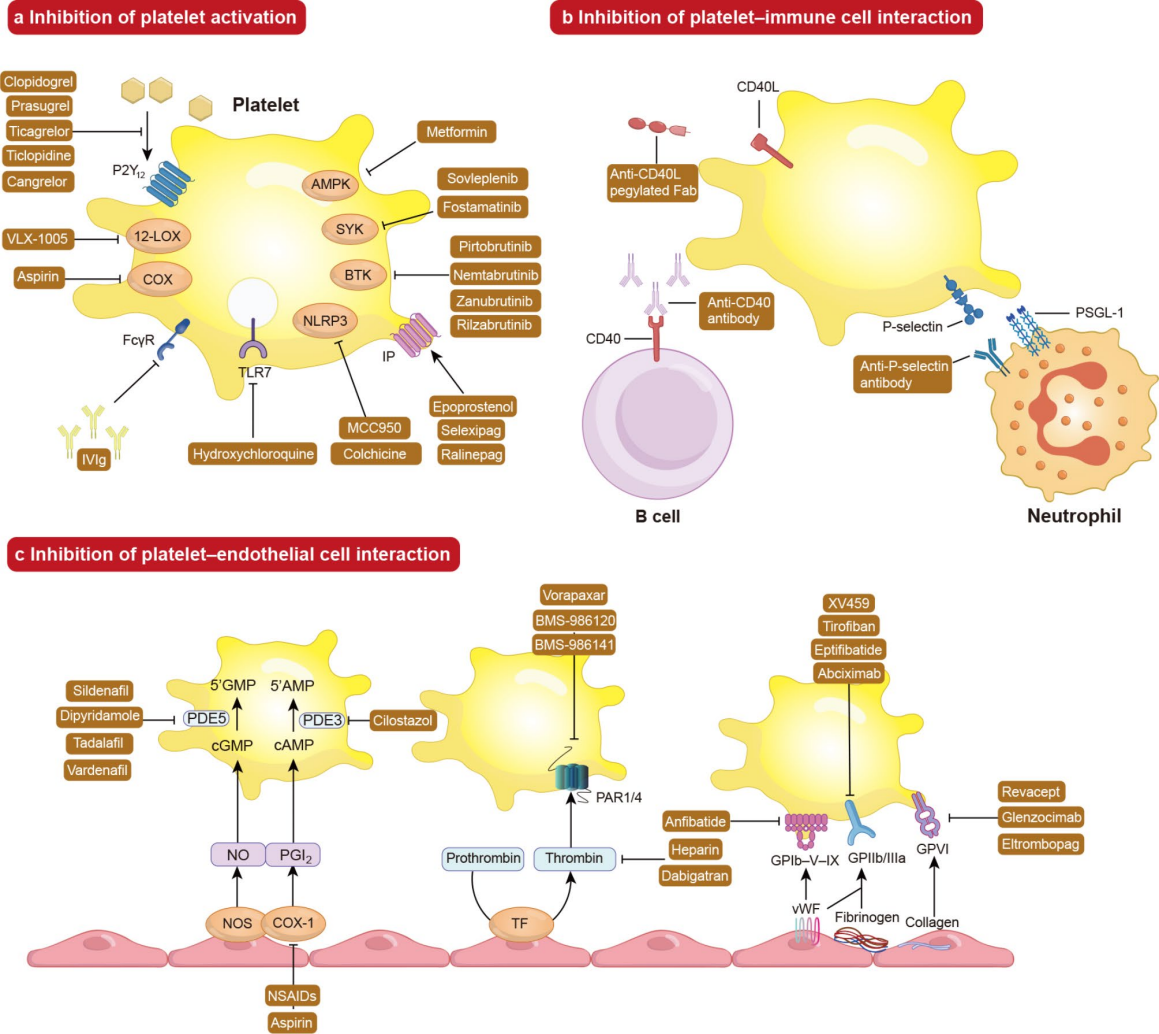
Clinical anti-platelet strategies to target the immune system. These strategies include targeting platelet activation, platelet-immune cell interaction, and platelet-endothelial cell interaction. (**a**) Targeting platelet activation. Platelet activation can be inhibited by various methods such as inhibiting agonist engagement. This includes targeting ADP binding to its receptor P2Y_12_ with medications like ticlopidine, clopidogrel, prasugrel, ticagrelor and cangrelor. Other medications include inhibiting platelet 12-LOX activity using VLX-1005, inhibiting platelet COX activity using aspirin, inhibiting FcγR activation using high-dose IVIg, inhibiting TLR7 activation using hydroxychloroquine, inhibiting the NNLRP3 inflammasome with colchicine and MCC950, inhibiting IP receptor with medications like epoprostenol, selexipag and ralinepag and inhibiting intracellular signaling pathways including inhibiting AMPK activation with metformin, inhibiting SYK activation with fostamatinib and sovleplenib and inhibiting (BTK activation with medications like pirtobrutinib, nemtabrutinib, zanubrutinib, and rilzabrutinib. (**b**) Targeting platelet-immune cell interaction. The interactions of platelet-immune cell are suppressed by affecting specific ligand-receptor pairs that mediate these interactions. For example, antibodies inhibit P-selectin binding to PSGL1 on neutrophils and other immune cells, while antibodies targeting CD40 or CD40L can inhibit interactions with B cells. (**c**) Targeting platelet-endothelial cell interaction. Activated NOS and COX-1 increase the synthesis of NO and PGI_2_ as well as intracellular contents of cAMP and cGMP. Medications like aspirin and NSAIDs act as COX-1 inhibitors to prevent COX-1-induced increased production of PGI_2_ and intracellular levels of cAMP. PDE5 inhibitors such as sildenafil, vardenafil, tadalafil and vardenafil limit the hydrolysis of cGMP to inactive 5’GMP. PDE3 inhibitors like cilostazol limit catalyzing the hydrolysis of cAMP to inactive 5’AMP. Prothrombin hydrolyzed activation into thrombin by the TF. Thrombin, activated by TF, can be blocked by medications like heparin and dabigatran. PAR1/4 inhibitors like vorapaxar, BMS-986120 and BMS-986141, which are principal thrombin receptors in platelets. GPIb-V-IX inhibitors like anfibatide inhibit the binding domain of vWF. GPVI inhibitors include revacept, glenzocimab, and eltrombopag. GPIIbIIIa inhibitors such as tirofiban, eptifibatide, abciximab, and XV459 decrease platelet activity by competing with fibrinogen and vWF

Platelets regulate immune responses by interacting with immune cells in immune-mediated inflammatory diseases [24]. They have the ability to modify immune cells and influence the behavior of their binding partners during platelet-immune cell interactions [12]. The comprehension of the communication between platelets and immune cells has broadened and now includes their interactions with both innate and adaptive immune cells (Fig. 3).

#### Innate immune cells

There is growing evidence that platelets play roles beyond their traditional functions in immune-mediated inflammatory diseases. Platelets interact with, stimulate and regulate cells of the innate immune system such as neutrophils, monocytes/macrophages, DCs, Natural Killer (NK) cells and Red blood cells (RBCs).

##### Neutrophils

The interaction between platelets and neutrophils is involved in the pathogenesis of immune-mediated inflammatory diseases, such as bacterial endotoxemia and ischemic stroke [127]. Platelets can actively adhere to neutrophils, accelerating tissue injury and autoantigen release via immunogenic cell death processes, especially NETosis or ferroptosis [128]. During bacterial endotoxemia, platelets express TLR4 stimulated by LPS, causing platelets to adhere to neutrophils, such as P-selectin exposure and aggregation, but no significant platelet activation [129]. Importantly, neutrophils aggregated with platelets exhibit active phenotypes, with increased expression of CD11b and a stronger oxidative burst caused by hypoxia-reoxygenation. This indicates that the adhesion of platelet to neutrophils triggers an outside-in signaling pathway that influences the phenotypic characteristics [130]. In ischemic stroke, neutrophils bind platelets through P-selectin and GPIbα and lead to platelet phosphatidylserine. Inhibition of procoagulant platelets decreases circulating platelet-neutrophil aggregates and thereby reduces infarct size. Platelet binding also alters neutrophil function, which contributes to the injury associated with ischemic stroke. This includes inducing the release of NETs, which are neurotoxic and pro-thrombotic, leading to impaired stroke outcomes [131].

The formation of platelet-NET complexes is a main functional disorder that results in thrombo-inflammation [132]. Neutrophils are recruited to inflammatory blood vessel walls via interactions with endothelial-attached platelets or the production of platelet-neutrophil aggregates [133]. Platelets bind to endothelium and neutrophils with endothelial activation and subendothelial matrix exposure. P-selectin binds to PSGL-1 in a Ca2^+^-dependent manner, whereas GPIb binds to neutrophil Mac-1. This facilitates the binding of CD40 on neutrophils and lymphocyte function-associated antigen-1 (LFA-1) to intercellular adhesion molecule (ICAM) 2 on platelets. These interactions promote coagulation that is dependent on NET formation and convert fibrinogen into fibrin in thrombo-inflammation [134].

NETosis is triggered by the interactions of P-selectin and PSGL1, indicating potential connection between platelet-neutrophil aggregation, thrombo-inflammation, and immunity system [135]. Antiphospholipid antibodies activate neutrophils, increase TF expression, and enhance susceptibility to NETosis, resulting in elevated arterial or phlebothrombosis in APS mice [136]. Notably, the prothrombotic phenotype can be reversed by inhibiting PSGL1 [137]. Additionally, in vivo oxidation of platelet-derived HMGB1 promotes NETosis and thrombo-inflammation in mice [138, 139]. This suggests that antiphospholipid antibodies may activate platelets, contributing to platelet-neutrophil aggregation, NETosis, and ultimately thrombo-inflammation.

Activated platelets via GPVI, which are inhibited by antiplatelet antibodies, boost neutrophil recruitment to the inflammatory site during immune complex-mediated inflammation in mice. Moreover, the heightened degranulation and subsequent release of elastase and matrix metalloproteases by neutrophils lead to tissue injury [140]. With further studies on the mechanism of interaction between platelet and neutrophil, treatments aimed at disrupting the platelet-neutrophil junction may become possible in the development of thrombo-inflammation.

##### Monocytes/Macrophages

Monocytes express adhesion molecules like PSGL1 and engage in interactions with platelet activation in immune-mediated inflammatory diseases [141]. The interaction between platelet P-selectin and monocyte PSGL-1 increases the adhesive capabilities of monocytes by upregulating the expression of β-integrins and adhesion to fibronectin, vascular cellular adhesion molecule-1 (VCAM-1) and ICAM-1 as well as enhancing transendothelial migration [142]. The interactions between platelets and monocytes are mainly mediated by through P-selectin and PSGL-1 as well as CD40L and CD40. Activated platelets also regulate monocyte cytokine production, which in turn promotes the differentiation of monocytes towards a pro-inflammatory DC phenotype with increased production of inflammatory cytokines such as CCL2, TNF-α, IL-1β and NF-κB. It is noteworthy that immunomodulatory substances like transforming growth factor-β (TGF-β) are also present in significant amounts in platelets. Moreover, several investigations indicate that monocytes exposed to platelet secretomes or lysates cause DCs with insufficient immunostimulatory activities to differentiate. Both of this pathway suggest that the interaction of monocytes and platelet contributes to the release of pro-inflammatory cytokines and immunomodulatory substances, finally lead to thrombo-inflammation [143].

Monocytes can differentiate into macrophages. Macrophages play a dual role in the pathophysiology of many immunologically mediated inflammatory disorders. They perform the dual roles of antigen-presenting cells, which activate the adaptive immune reaction, and effector cells, which destroy platelets. Macrophages generated from monocytes show that antibodies identify platelets to initiate an immune response, and that platelet absorption by macrophages is dependent on FcγRI ligation. FcγRI activation stimulates the production of the potent vasoactive mediator, platelet-activating factor, which causes an anaphylactic response [144].

##### DCs

Monocytes have the ability to differentiate into DCs in both appearance and functionality. Monocyte-derived dendritic cells (moDCs) are involved in the autoimmune response, phagocytosis of apoptotic platelets, and stimulation of specific T cells. moDCs express high levels of the costimulatory molecules CD86 and CD80 and produce substantial amounts of IL-12 in ITP. Additionally, pDCs as a subset of DCs have been implicated in the pathogenesis of immune-mediated inflammatory diseases [145]. Platelets can activate pDCs to secrete IFNα via the CD40L-CD40 axis in SLE. Targeting or mimicking these interactions to limit atherosclerosis or thrombo-inflammation by blunting myeloid recruitment, boosting regulatory T cells, inhibiting platelet activity [146]. The CD40 receptor, which belongs to the TNF-α family, is expressed by dendritic cells, B cells, and macrophages. CD40 activation by CD40L is essential for the autoimmune response [147]. These findings underscore the significance of platelets and the platelet-derived CD40L-CD40 axis in autoimmunity, which inhibit not only platelet aggregation and endothelial activation, but also vascular repair angiogenesis.

##### NK cells

The number of NK cells in the circulating blood of patients with ITP increases, which is correlated with the severity of the disease, but their functional activity has not been assessed [148]. Recent research indicated that circulating NK cell numbers are identical in ITP and control groups, maintaining cytotoxic capability, but showing lower production of IFN-γ in ITP. Importantly, it has been demonstrated that NK cells, unlike CD8^+^ T cells, do not participate in platelet lysis [149]. In patients with severe COVID-19 disease symptoms, a study points to the emergence of an NK cell subset with a platelet gene signature and aggregating of NK cells with CD42a^+^CD62P^+^activated platelets, which contributes to the development of thrombo-inflammation [150]. Therefore, further investigation is needed to fully understand how NK cells contribute to the development of various immune-mediated inflammatory diseases, particularly their potential role in regulating platelet production.

##### Red blood cells

RBCs bind to platelet, which promotes the development of sickle cell diseases. RBCs are involved in platelet-driven contraction of clots and thrombi that results in formation of a tightly packed array of polyhedral erythrocytes, or polyhedrocytes, which comprises a nearly impermeable barrier that is important for hemostasis and thrombo-inflammation [151]. Activated platelets up-regulate the expression of P-selectin, which interacts with PSGL-1 to connect platelets to different white RBCs. In addition, platelets produce white blood cell stimulating molecules that promote platelet-RCBs interactions and white blood cell activation in ITP [152]. All these phenomena display that platelet-RCBs interactions maybe play a key role in thrombo-inflammation.

#### Adaptive immune cells

Platelets play a crucial role in various immune-mediated inflammatory diseases by affecting multiple aspects of adaptive immunity, including antigen trafficking and presentation facilitated by DCs, as well as T and B cell signaling, maturation, and polarization.

##### T cell

The function of T_reg_ cells is compromised by low active platelet counts, and treatment that normalizes the platelet compartment also restores T_reg_ cell functionality [153]. The maturation of DCs and the promotion of antigen presentation by platelets affect CD4^+^ T cell polarization, an essential stage in determining the subsequent immune response [154]. Platelets through FcγRIIA receptors, may recognize immunological complexes and get activated by them, which may stimulate antibody responses [155].

While PSGL1 is expressed by all human CD4^+^ T cell subsets, only Treg cells and T follicular regulatory cells carry PSGL1 that has the sLe^X^ motif. PSGL1 activates and phosphorylates spleen tyrosine kinase (SYK) during P-selectin interaction with sLe^X^-containing PSGL1, resulting in SYK-dependent calcium signaling. P-selectin interaction in human T_reg_ cells rewires their transcriptional program, leading to TGF-β axis downregulation and ultimately a decrease in T follicular regulatory cells and T_reg_ cell’s ability to suppress the immune system.

Elevated platelet-CD4^+^ T cell aggregation with a larger T follicular helper cell compartment, enlarged germinal centers in lymph nodes, and higher autoantibody titers are characteristics that set these phenotypes apart. The significance of the P-selectin-PSGL1 connection in the pathophysiology of SLE is demonstrated by the fact that inhibiting the P-selectin-PSGL1 axis with a monoclonal antibody to P-selectin causes a milder form of lupus-like illness [156]. A platelet-specific PTEN deficit, which results in intrinsic platelet activation, can lead to fatal autoimmune and lymphoproliferative disorders [157].

##### B cell

The communications of platelet-B cell are more uncommon compared to contacts between platelets and other types of immune cell because B cells are absence of PSGL1 [158]. Individuals with SLE have higher contents of platelet-B cell aggregation, which are associated with blood contents of immunoglobulins and preswitched memory B cells [159]. The process of co-culturing human B cells and platelets increasing immunoglobulin synthesis and antibody type switch is mediated by the connection of CD40L and CD40 [160]. The primary source of bloodstream CD40L, either in soluble or surface form, is platelets, and the establishment of a B cell response dependent on T cells is significantly facilitated by the CD40L-CD40 axis [161]. Besides, increased platelet CD40L expression enhances the pathogenic antibodies synthesis to GPIIb/IIIa [162].

## Potential therapeutic modulations

Beyond their function in hemostasis and thrombosis, platelets communicate with the endothelium and immune cells in the bloodstream to promote thrombo-related inflammation and immunothrombosis. Therefore, developing clinical anti-platelet medications based on our understanding of the pathogenesis is a promising therapeutic strategy for immune-mediated inflammatory diseases (Table 1).

### Targeting platelet activation

Numerous clinical investigations have shown that platelet activation occurs in immune-mediated inflammatory disorders via the mechanisms discussed in the preceding section. Thus, inhibiting platelet activation is seen as a therapeutic strategy.

#### P2Y12 receptor inhibitors

Ticlopidine and clopidogrel are P2Y_12_ receptor inhibitors that are variable and irreversible, while prasugrel, ticagrelor and cangrelor are alternative P2Y_12_ receptor inhibitors [163]. Ticlopidine, a derivative of thienopyridine, is a prodrug that can be taken orally and has to be converted by the liver into an active metabolite to be effective. Ticlopidine treatment improves the immune and inflammatory responses mediated by B and T lymphocytes (NCT02244710 and NCT02428374) [164, 165]. During sepsis mice, ticlopidine decreases circulating white blood cells and platelet activation and platelet-leukocyte interactions [166]. Similar to ticlopidine, clopidogrel is a prodrug of thienopyridines that must be bioconverted by hepatic cytochrome P450 (CYP450) isoenzymes into an active metabolite. The active metabolite then binds to platelet P2Y_12_ receptors in a selective and irreversible manner, preventing ADP-stimulated platelet activation [167]. For example, a proof-of-concept study using the P2Y_12_ inhibitor clopidogrel for 12 weeks in patients with SLE reveals that the mean platelet volume, P-selectin-positive platelets, and the number of activated CD40L^+^ platelets all significantly decrease after starting clopidogrel treatment, yet these changes did not persist over time [168]. Clopidogrel has an anti-inflammatory and antithrombotic property in patients with chronic HIV infection due to the inhibition of thrombogenicity and sCD14 [169]. Besides, clopidogrel-treated mice show a decrease in aggregation of platelets and CD4^+^ T cells, which protect against the adverse outcomes of sepsis [170]. Platelets play a crucial role in various immune-mediated inflammatory diseases, given that their pathogenesis involves inflammation, platelet reactivity and innate immune activation.

Prasugrel, ticagrelor, and cangrelor are potent platelet inhibitors that may be beneficial in various immune-mediated inflammatory diseases. Prasugrel is a thienopyridine and a prodrug. Prasugrel abolishes the effects of platelets on CD4^+^ T-cells with similar levels of pro-inflammatory cytokines IFN-γ and cell numbers to T-cells stimulated [171]. Ticagrelor, in comparison to clopidogrel, exhibits stronger antiplatelet effects as it binds directly and reversibly to P2Y_12_ receptors. Studies have shown that ticagrelor, compared to clopidogrel, improves inflammatory parameters like neutrophil-to-lymphocyte ratio, monocyte-to-high-density lipoprotein ratio, platelet-to-lymphocyte ratio, and systemic immune-inflammation index in patients with percutaneous coronary intervention-treated acute coronary syndrome [172]. Ticagrelor interrupts platelet-neutrophil interaction by attenuating NETs induced by polyP in thrombo-inflammation [173]. In Alzheimer’s disease mice, cangrelor may reverse Aβ_1−42_-induced cognition deficits by inhibiting oxidative stress, neuroinflammation, and synaptic dysfunction mediated by nuclear factor E2-related factor 2 (Nrf2)/heme oxygenase 1 (HO-1) and NF-κB signaling [174].

#### COX-1 inhibitors

Aspirin, or acetylsalicylic acid, is a unique nonsteroidal anti-inflammatory medication used for the treatment of acute inflammation. High doses of aspirin exhibit anti-inflammatory functions by inhibiting cyclooxygenase-1 (COX-1) and proinflammatory signaling pathways, including NF-ĸB. Lower doses of aspirin demonstrate cardioprotective functions by promoting the synthesis of proresolution 15-epi-lipoxin A (4) and inhibiting thromboxane (Tx) B (2), a prothrombotic eicosanoid also involved in polymorphonuclear leukocyte trafficking [175]. For example, aspirin has the unique ability to stimulate the synthesis of 15-epi-lipoxin A4, induce the release of anti-adhesive NO to exert its protective effect, thereby inhibiting leukocyte/endothelial cell interactions and subsequent extravascular leukocyte migration [175, 176]. However, in phase 2b trial, aspirin therapy in acute inflammatory lung injury [177] doesn’t significantly reduce the incidence of acute respiratory distress syndrome (ARDS) (NCT01504867) [178]. Besides, aspirin has less of an inhibitory impact on platelet-leukocyte aggregation and surface expression of P-selectin and CD40L than P2Y_12_ inhibitors do. In individuals with existing coronary artery disease, aspirin monotherapy may not be as effective as P2Y_12_ inhibitor monotherapy for long-term secondary prevention. Since the reaction to aspirin is reduced in individuals with SLE and coronary artery disease, it is unclear whether aspirin monotherapy has a significant immunotherapy effect [179].

Nonsteroidal anti-inflammatory drugs (NSAIDs) are COX-1 inhibitors used worldwide to treat different immune-mediated inflammatory diseases. These drugs work by irreversibly acetylating COX-1 and COX-2 in endothelial cells, thereby inhibiting the production of prostaglandins such as PGI_2_ [180]. NSAIDs, such as ibuprofen and dipyrone (metamizole), can transiently inhibit aspirin from acetylating COX-1 at serine 530 through reversible competition [181]. A narrative review suggests that ibuprofen may play a role in reducing excessive inflammation or cytokine release by inhibiting the transcription factor NF-kB in COVID-19 patients [182]. Dipyrone medication downregulates the expression of Th2 and TNF-α in human monocytes [183].

#### 12-LOX inhibitors

Another type of oxygenase expressed by platelets is 12(S)-lipoxygenase (12-LOX), which can convert arachidonic acid (AA) into a prothrombotic oxylipin [184]. VLX-1005 is a new 12-LOX inhibitor with excellent selectivity that inhibits platelet activation and reduces thrombus formation. Recent discoveries have revealed that the binding site of VLX-1005 is physically distant from the active site, contradicting the initial hypothesis that VLX-1005 binds to 12-LOX at the active site and prevents AA from binding [185]. VLX-1005 appears to have minimal effects on hemostasis, lowering the risk of bleeding. However, it does seem to have the ability to bind to 12-LOX concurrently [186]. The potential of VLX-1005 to prevent thrombosis in immune-mediated platelet activation, such as heparin-induced thrombocytopenia (HIT), is being evaluated. Inhibiting 12-LOX has been shown to be an effective strategy for preventing immune-mediated platelet activation and the development of HIT. For example, a recent study demonstrated that inhibiting 12-LOX with VLX-1005 in a human and mouse model of HIT decreases platelet activation downstream of FcγRIIA and protease-activated receptor (PAR4). This indicates that targeting 12-LOX could be a promising clinical approach for immune-mediated inflammatory diseases [187].

#### Intravenous immunoglobulin and SYK inhibitors

Intravenous immunoglobulin (IVIg)-mediated platelet activation is a promising treatment [46]. High-dose IVIg inhibits FcγRIIA in immune-mediated inflammatory diseases [188]. For example, high-dose IVIg therapy reduces the inflammatory response of myeloid DCs in humans by Th2 cytokine-mediated downregulation of FcγRIIA and IFN-γR2, rather than overexpression of FcγRIIb. This work implies that this cascade is initiated by stimulating IL-33 production, which seems to be DC-specific intercellular adhesion molecule-grabbing and nonintegrin independent [189]. Besides, IVIg has the ability to improve thrombin-induced platelet activation and enhance thrombin generation in a prospective study of 23 children with primary ITP, demonstrating that besides increasing platelet counts IVIg can be efficacious on the level of thrombin-induced platelet activation and coagulation support [190]. Immunoglobulin G antibodies also coordinate immune effector responses by interacting with effector cells via Fcγ receptors [191]. FcγRIIA communicates partly through SYK. Fostamatinib as a SYK inhibitor is authorized for treat in patients with ITP and reduce platelet phagocytosis by splenic macrophages, which inhibit platelet activation [192]. Fostamatinib is a novel therapy for ITP that targets a key pathophysiological process. According to phase III placebo-controlled studies, fostamatinib demonstrates clinically significant responses in ITP patients with chronic immune thrombocytopenia (NCT02612558) [193]. Moreover, in a phase 3 global trial, fostamatinib exhibits a significant effect for individual in Western regions, without new safety signals identified [194]. Sovleplenib is also a SYK inhibitor, shows a promising durable response in patients with primary immune thrombocytopenia [195].

#### TLR7 inhibitors

TLR inhibitors may be a promising strategy in immune-mediated inflammatory diseases. Hydroxychloroquine (HCQ) is the primary therapy for SLE and other connective tissue diseases [196]. HCQ restricts endosome acidification, resulting in impaired endosomal TLR7 activation and IFN-α production [197]. Additionally, it has been demonstrated that HCQ decreases platelet activation, when stimulated ex vivo with ADP or antiphospholipid antibodies. Platelet activation and the expression of P-selectin are reduced in HCQ-treated individuals. These effects support the extensive reference associating HCQ medication with reduced thrombosis risk in SLE (NCT00413361) and APS (NCT01034137) patients [198, 199]. Furthermore, interactions with CD8^+^ T lymphocytes are limited because HCQ ‘strips’ MHC class I molecules from the platelet membrane. Moreover, HCQ medication is reported to result in clinically significant reductions in vascular inflammation in individuals with RA participating in a randomized active comparator trial (NCT02374021) [200]. And HCQ treatment decreases platelet TLR expression and function together with decreased circulating platelet-monocyte aggregates, HMGB1 levels and platelet vWF release in the inflammatory state of SLE [201].

#### NLRP3 and BTK inhibitors

Colchicine, an NLRP3 inhibitor, is commonly recommended for individuals with immune-mediated inflammatory diseases including gout, SLE, and pericarditis. Colchicine prevents microtubule aggregation and NLRP3 inflammasome activation [202]. Surprisingly, multiple clinical studies have linked colchicine use to a reduced risk of cardiovascular disease. For example, among individuals with chronic coronary disease, the risk of cardiovascular events significantly lower in those who received colchicine compared to those who received a placebo (ACTRN12614000093684 and NCT02551094) [203–205]. This effect is attributed to various mechanisms, including inhibiting inflammasome aggregation and the release of IL-1β, as well as directly affecting platelets. Besides, colchicine inhibits activated platelets and the formation of platelet-neutrophil and platelet-monocyte aggregation in healthy individuals [206]. In the cecal-ligation puncture rat model of sepsis, MCC950 is another NLRP3 inhibitor, attenuates NLRP3 activation in platelets and decrease the levels of NLRP3 inflammasome associated cytokines [207].

Moreover, it has been found that pharmacologically inhibiting Bruton tyrosine kinase (BTK) is an effective way to block the NLRP3 inflammasome, which may consequently prevent platelet activation. Pirtobrutinib, a highly selective, noncovalent (reversible) BTK inhibitor, is designed to reestablish BTK inhibition. In a phase 1–2 trial, pirtobrutinib demonstrates effectiveness in patients with heavily pretreated chronic lymphocytic leukemia (CLL) or small lymphocytic lymphoma (SLL) who have received a covalent BTK inhibitor. The most common adverse events are infections, bleeding, and neutropenia (NCT03740529) [208]. Nemtabrutinib also inhibits the C481S mutant BTK, indicating its safety and potential benefit for individuals with relapsed or refractory B-cell malignancies [209]. As a more selective BTK inhibitor, zanubrutinib is used to treat relapsed or unresponsive SLL or CLL. Compared to ibrutinib, zanubrutinib has a noticeably longer progression-free survival through the inhibition of unusual and healthy B cells. However, zanubrutinib is linked to a lower number of adverse cardiac events (NCT03734016) [210]. Rilzabrutinib, is also inhibitor of BTK, increase platelet counts in patients with immune thrombocytopenia by means of decreasing macrophage (Fcγ receptor)-mediated platelet destruction and reducing production of pathogenic autoantibodies [211].

#### Prostacyclin receptor agonists

The prostacyclin (IP) receptor belongs to the prostaglandin receptor family of G-protein-coupled receptors (GPCRs). Prostaglandin I2 (PGI_2_), the principal IP receptor agonist, is widely recognized for its strong vasodilator properties, which have led to its application in the management of pulmonary arterial hypertension (PAH) [212] and primary pulmonary hypertension (PPH) [213]. The first licensed treatment for PAH is epoprostenol, a synthetic analog of PGI_2_, which significantly enhances T_reg_ function and increases the survival rates of patients with PAH and PPH (NCT03081052) [214]. In autoimmune encephalomyelitis and systemic sclerosis, PGI_2_ suppresses Th1 and Th2 immune responses and stimulates Th17 differentiation [215, 216]. The two primary IP analogs that enhance blood stability are ralinepag and selexipag. In PAH patients on either monotherapy or dual combination baseline treatment, a phase 2 study (NCT02279160) shows that ralinepag significantly enhances T_reg_ function and decreases pulmonary vascular resistance compared to placebo [217]. In individuals with PAH, selexipag upregulates in macrophages and reduces the risk of mortality events, which are the major composite endpoint of death or PAH-related complications (NCT01106014) [218, 219]. Additionally, in patients with chronic thromboembolic pulmonary hypertension, selexipag significantly improves various hemodynamic measures and pulmonary vascular resistance in this placebo-controlled study [220].

#### AMPK inhibitors

AMP-activated protein kinase (AMPK) inhibitor has shown promising antiplatelet effects [221]. Metformin, a medication commonly used to treat type 2 diabetes, works by activating the AMPK metabolic regulator. Its ability to influence immune cell metabolism has been demonstrated in animal models and in promising proof-of-concept trials involving patients with SLE or multiple sclerosis [222, 223]. It is interesting to note that metformin inhibits platelet activation in vitro and suppresses the release of mitochondrial DNA (mtDNA), a process that contributes to the etiology of SLE and other immune-mediated inflammatory diseases. Metformin reduces the risk of arterial and venous thrombosis in patients with diabetes mellitus and in vivo models, supporting its beneficial clinical effects in immune-mediated inflammatory diseases with elevated cardiovascular risk factors (NCT00006305) [224, 225]. Metformin also blocks LPS-induced and ATP-dependent mtDNA synthesis and generation of oxidized mtDNA, an NLRP3 ligand, in ARDS [226]. In hidradenitis suppurativa, metformin significantly decreases lymphocytes, monocyte-lymphocyte ratio, neutrophil-lymphocyte ratio, platelet-lymphocyte ratio, serum adipokines, and immune mediators [227].

### Targeting platelet-immune cell interaction

Targeting platelet-immune cell interaction in multiple immune-mediated inflammatory diseases is a promising strategy to treat such conditions. Numerous clinical trials indicate that the regulation of platelet-immune cell interaction is currently under investigation as a therapeutic approach.

#### Anti-P-selectin antibody

P-selectin blocking is a potentially effective treatment for immune-mediated inflammatory diseases because it inhibits the formation of platelet-leukocyte aggregates in humans [228]. In mice, a P-selectin-blocking antibody activates monocytes and neutrophils acceleration and promotes venous thrombosis resolution due to reduced infiltration and activation of innate immune cells at the site of thrombus formation, which prevents early thrombus stabilization and facilitates fibrinolysis [229]. Crizanlizumab is a human monoclonal P-selectin antibody used for patients with COVID-19 and sickle cell disease. It binds to P-selectin and inhibits its interaction with PSGL-1 (NCT04505774) [230]. Crizanlizumab has a strong safety profile based on numerous trials and reduces the occurrence of vaso-occlusive crises in these individuals. Importantly, there is no observed elevated risk of infection, which might have been expected given that platelets promote leukocyte migration to combat infections [231, 232].

Some antibodies to double-stranded DNA, proteinuria and kidney damage are common in SLE, especially in individuals with lupus nephritis. For instance, impaired DNASE1L3 activity occurs when P-selectin is blocked by a monoclonal antibody, which is a prevalent non-genetic mechanism that enhances anti-dsDNA autoreactivity and restores T_reg_ cell functions [233]. Furthermore, various studies indicate that P-selectin inhibition reduces hypoxia in renal tissue, improves proteinuria and mitigates kidney damage in lupus-prone MRL/faslpr mice [234]. Moreover, a PSGL1 glycomimetic, another P-selectin inhibitor, is a potential medication candidate that prevents venous thrombosis without increasing the risk of bleeding [141]. P-selectin inhibition has multiple immunological effects, does not induce immunosuppression, and may prevent thrombosis, making it a promising therapeutic approach for immune-mediated inflammatory diseases.

#### Anti-CD40L antibody and antigen-binding fragment

Monoclonal antibodies against CD40L have also been investigated in SLE patients. However, these antibodies have been associated with significant thrombotic events, likely due to antibody-mediated crosslinking of CD40L on activated platelets, which then triggers further platelet activation and aggregation through FcγRIIA ligation [235]. Recent studies on autoimmune diseases have explored novel anti-CD40L molecules that do not bind to FcγRIIA, such as dapirolizumab, an anti-CD40L antigen-binding fragment (Fab) with a pegylated tail [236], or a monoclonal antibody that lacks the ability to bind to the Fc receptor [237]. Dapirolizumab therapy don’t achieve the primary goal in the phase II trial. However, it appears to be well tolerated. Several clinical and immunological markers of disease activity have shown improvements in the patients [236]. As a result, dapirolizumab is currently being evaluated in a phase III study, expected to be completed in 2024 (NCT04294667). Other monoclonal antibodies, such as BI 655,064, have been developed to target CD40 expressed by B cells and T cells. The study is unable to demonstrate a dose-response relationship for the primary endpoint of complete renal response (NCT02770170) [238]. Besides, in *vivo* study, CDP7657, an anti-CD40L antibody lacking an Fc domain, inhibits CD40L-dependent immune responses without thrombotic complications, nevertheless retain pharmacological activity and support the investigation of CDP7657 as a potential therapy for SLEs and other autoimmune diseases [239].

### Targeting platelet-endothelial cell interaction

The platelet-endothelial cell interaction is associated with the immune response, which may be a promising strategy to treat multiple immune-mediated inflammatory diseases. Many clinical trials have shown that regulating platelet-endothelial cell interaction can ameliorate the severity of immune-mediated inflammatory diseases.

#### PDEs inhibitors

Phosphodiesterases (PDEs) are enzymes that restrict intracellular levels of these cyclic nucleotides by catalyzing the hydrolysis of cyclic adenosine monophosphate (cAMP) and cyclic guanosine monophosphate (cGMP) into inactive 5’adenosine monophosphate (5’AMP) and 5’guanosine monophosphate (5’GMP), respectively. Cyclic adenosine monophosphate (cAMP) and cyclic guanosine monophosphate (cGMP) are essential for intracellular secondary messengers that control platelet activity [240]. In endothelial cells, activated nitric oxide synthase (NOS), raising nitric oxide increases, intracellular levels of cAMP and cGMP. This subsequently triggers the cAMP-PKA/EPAC-dependent and cGMP-PKG-dependent protein kinase I pathways [241], respectively. EPAC is another cAMP effector which inhibit P_2_ × _1_ receptors and platelet aggregation [242]. Epac1^−/−^mouse have increased size of circulating platelets [243]. Therefore, one effective strategy for reducing platelet hyperactivity is to target platelet PDEs. PDEs expressed in platelets include PDE3 and PDE5. PDE3 prefers cAMP as a substrate, whereas PDE5 prefers cGMP [241].

Cilostazol is a potent PDE3 inhibitor that reduces platelet reactivity and aggregation by increasing intracellular cAMP levels. Cilostazol shows promise as a drug against COVID-19 by inhibiting both the main protease and Spike glycoprotein, reinforcing its potential as a therapeutic approach for COVID-19 [244]. Dipyridamole-induced production of nitrite/NO significantly downregulates the expression of innate immunity genes such as IL-10, TLR1 and TLR10, while promoting vascular growth in ischemic tissues [245]. In zebrafish, cilostazol treatments lead to a subtle yet significant effect on reducing the aggregation of Mpx-expressing neutrophil at the lesion site, but did not affect the immediate injury-induced recruitment and retention of Mpeg1-expressing macrophages [246].

PDE5 inhibitors, commonly used in clinical settings, include sildenafil, vardenafil, and tadalafil. These drugs can reduce platelet reactivity by increasing intracellular cGMP levels [247]. In humans, treatment with sildenafil affect the innate and adaptive immune system by regulating angiogenesis, platelet activation, proliferation of regulatory T cells, and production of proinflammatory cytokines TNF-α and IL-1β, as well as autoantibodies in animals and patients [248, 249]. Vardenafil has been proven to be effective and down-regulates M1 macrophage pro-inflammatory markers (NOS-2 and TNF-α) in individuals with pulmonary arterial hypertension in a randomized, double-blind, placebo-controlled study [250]. Furthermore, tadalafil can reverse tumor-induced immunosuppression and promote tumor immunity in patients with head and neck squamous cell carcinoma (HNSCC) by reducing both myeloid-derived suppressor cells and T_reg_ concentrations in the blood and in the tumor [251]. In a two-arm multi-institutional neoadjuvant randomized trial, tadalafil alters the immune microenvironment, as evidenced by transcriptome data identifying enriched B- and natural killer cell gene sets in the tumor and augmented effector T cells in the periphery in any-stage resectable HNSCC (NCT03238365) [252].

#### PAR-1/4 inhibitors

PAR-1/4 is the primary thrombin receptor in the vasculature, and antagonists against this receptor are currently undergoing preclinical trials [253]. One of the PAR1 antagonists is vorapaxar. Vorapaxar significantly increased CD4 and CD8 T cells in treated HIV-associated inflammation and coagulopathy. However, vorapaxar has no effect on D-dimer concentrations in HIV-infected patients receiving stable antiretroviral therapy but at risk of poor outcomes [254].

The activation mechanisms of both PAR1 and PAR4 receptors are similar, but the thrombin affinity between the two receptors is significantly different, probably due to the N-terminal exodomain of PAR4 lacking a hirudin-like sequence present in PAR1. This sequence in PAR1 interacts with thrombin exosite 1, hindering the interaction between PAR4 and thrombin [255]. PAR4 antagonists, such as BMS-986,120 and BMS-986,141, are currently in different phases of preclinical and clinical development for vascular inflammation and traumatic brain injury. Both candidates significantly inhibit thrombin-induced inflammation in astrocytes through the Tab2/ERK/NF-κB signaling pathway, which have the potential to improve traumatic brain injury [256].

#### Thrombin blockers

Unfractionated heparin is a naturally occurring glycosaminoglycan that binds antithrombin III (ATIII), causing a conformational change that leads to a 1000-fold increase in ATIII’s capacity to inhibit thrombin, FXa, and other coagulation serine proteases [257]. Heparin treatment demonstrates a comparable safety profile and reduces the occurrence of catheter-related bloodstream infections in participants undergoing hemodialysis via central venous catheter (NCT02651428) [258]. However, heparin treatment increases the risk of bleeding and does not reduce mortality, organ damage and HIT. HIT is a severe drug reaction that results in a decrease in platelet count and a high risk of thrombosis. Patients with HIT produce pathogenic immunoglobulin G antibodies that bind to complexes of PF4 and heparin. The response to HIT involves a temporary loss of immune regulation for T-cells and marginal zone B-cells, but crucial findings are yet to be confirmed in human disease [259, 260]. Dabigatran anticoagulation, a specific thrombin inhibitor, counteracts the effects of thrombin in astrocytes by limiting the activation of PAR-1, thereby downregulating sphingosine kinases and disrupting sphingosine-1-phosphate receptor signaling in multiple sclerosis. This study sheds light on the connection between coagulation mechanisms and immune diseases of the central nervous system [261].

#### GPIb-V-IX inhibitors

The subunits GPIbα, GPIbβ, GPV, and GPIX make up the GPIb-V-IX complex in a 2:2:1:2 ratio [262]. vWF and thrombin, the two most well-known ligands of the GPIb-IX complex, bind to the platelet via GPIbα. The main function of GPIb is to bind vWF, which mediates the first stage of platelet adhesion by stopping the flow of platelets and allowing them to adhere to the extracellular matrix or endothelium at the site of damage [263]. Anfibatide is a non-enzymatic protein that resembles a C-type lectin extracted from the venom of the Agkistrodon acutus snake. It binds to the GPIbα subunit of the GPIb-V-IX complex in a competitive manner, preventing vWF from binding. Anfibatide prevents neutrophil NETosis and NET formation dependent on platelets and vWF in the pathogenesis of immune-mediated thrombotic thrombocytopenic purpura [264]. Anfibatide has anti-thrombo-inflammatory properties upon stroke by decreasing the number of microthrombi formation and reducing the expression of MAC-1 and P-selectin in the treatment of ischemic stroke [265].

#### GPVI inhibitors

GPVI is a platelet-specific receptor expressed on the surface of circulating platelets that binds to collagen, the primary prothrombotic component of a plaque [266]. When GPVI, the principal platelet collagen receptor, is activated, platelets aggregate and form clots. GPVI plays an important role in inflammation by enhancing neutrophil-damaging activities while supporting the activation and hemostatic adhesion of single platelets to neutrophil-induced vascular breaches [140]. Revacept competes with endogenous GPVI for binding to both collagen and vWF, interfering with the platelet response to exposed collagen. According to a phase 2 randomized clinical study, revacept does not reduce myocardial damage in patients receiving percutaneous coronary intervention who have stable ischemic heart disease (NCT03312855) [267]. Additionally, revacept decreases the combined safety and effectiveness endpoint by blocking the collagen binding sites at the vascular lesion in individuals with symptomatic internal carotid artery stenosis, according to another phase II trial (NCT01645306) [268]. Glenzacimab, a humanized monoclonal antibody fragment designed to inhibit GPVI, is an alternative strategy for blocking the collagen-GPVI pathway. Glenzacimab is well tolerated in the phase 1b/2a study and may reduce mortality, cerebral hemorrhage, and major adverse effects. This study underscores the need for further investigation into glenzocimab-mediated therapeutic inhibition of GPVI in acute ischemic stroke patients [269]. Eltrombopag via its effect on the GPVI receptor expression and its soluble form, reduce bleeding manifestations and improve the quality of life of chronic and persistent ITP children independent of its effect on the platelet count [270].

#### GPIIbIIIa inhibitors

GPIIbIIIa inhibitors, also known as integrin αIIbβ3 inhibitors, are specific platelet receptors essential for both pathological thrombus formation and physiological hemostasis [271]. The FDA has approved three GPIIbIIIa inhibitors for clinical use: tirofiban, eptifibatide, and abciximab. They decrease platelet activity by competing with fibrinogen and vWF for integrin αIIbβ3 binding. Intravenous tirofiban has been associated with a higher likelihood of a favorable outcome in stroke patients compared to low-dose aspirin. However, tirofiban treatment is also more likely to lead to immune thrombocytopenia (ChiCTR2000029502) [272, 273]. Intravenous tirofiban treatment, as opposed to placebo, prior to endovascular therapy, does not significantly alter the degree of impairment at 90 days in patients with major artery occlusion acute ischemic stroke undergoing endovascular thrombectomy (ChiCTR-IOR-17014167) [274]. Eptifibatide and abciximab therapy are associated with immune-mediated thrombocytopenia with antibodies recognizing the αIIbβ3 receptor only in the presence of the drug [275]. XV459, a free acid form of roxifiban, blocks platelet activation/aggregation and significantly decrease levels of fibrinogen binding to human platelets, which show a potential benefit in the management of thrombotic thrombocytopenia produced by heparin and/or related glycosaminoglycans [276].

**Table 1:** Immunoregulation involved in clinical anti-platelet therapies

Modulation	Target	Candidate drug	Mechanism of action	Reference
Targeting platelet activation	P2Y_12_ receptor	Ticlopidine	Improving the immune and inflammatory responses mediated by B and T lymphocytes	[164, 165]
		Clopidogrel	Decreasing P-selectin-positive platelets, activated CD40L^+^ platelets and inhibiting sCD14	[168, 169]
		Prasugrel	Abolishing the effects of platelets on CD4^+^ T-cells and inhibiting pro-inflammatory cytokines IFN-γ	[171]
		Ticagrelor	Improving inflammatory parameters like neutrophil-to-lymphocyte ratio, monocyte-to-high-density lipoprotein ratio, platelet-to-lymphocyte ratio, and systemic immune-inflammation index	[172]
		Cangrelor	Inhibiting neuroinflammation mediated by Nrf2/HO-1 and NF-κB signaling	[174]
	COX-1	Aspirin	Triggering the synthesis of 15-epi-lipoxin A4 and inhibiting leukocyte/endothelial cell interactions	[175, 176, 178, 179]
		Ibuprofen	Inhibiting of the transcription factor NF-kB	[182]
		Dipyrone	Downregulating expression of Th2 and TNF-α	[183]
	12-LOX	VLX-1005	Decreasing platelet activation downstream of FcγRIIA and PAR4	[187]
	IVIg	IVIg	Reducing the inflammatory response of myeloid DCs by Th2 cytokine-mediated downregulation of FcγRIIA and IFN-γR2	[189]
	SYK	Fostamatinib	Blocking the immune signaling pathway of the FcγRIIA-SYK	[192, 193]
		Sovleplenib	Inhibiting SYK	[195]
	TLR	Hydroxychloroquine	Impairing endosomal TLR7 activation and IFN-α production and limiting the interactions with CD8^+^ T lymphocytes	[197–199]
	NLRP3	Colchicine	Inhibiting inflammasome aggregation and IL-1β production and reducing the quantity of platelet-neutrophil and platelet-monocyte aggregation	[206]
		MCC950	Attenuating NLRP3 activation in platelets and decrease the levels of NLRP3 inflammasome associated cytokines	[207]
	BTK	Pirtobrutinib	Inhibiting unusual and healthy B cells gather	[208]
		Nemtabrutinib		[209]
		Zanubrutinib		[210]
		Rilzabrutinib	Increasing platelet counts by decreasing macrophage (Fcγ receptor)-mediated platelet destruction and reducing production of pathogenic autoantibodies	[211]
	IP	Epoprostenol	Enhancing T_reg_ function	[214]
		Ralinepag		[217]
		Selexipag	Upregulating in macrophages	[218–220]
	AMPK	Metformin	Inhibiting NLRP3 inflammasome activation and IL-1β and IL-6 production	[226]
Targeting platelet-immune cell interaction	P-selectin	Crizanlizumab	Inhibiting the formation of platelet-leukocyte aggregates	[230–232]
	CD40L	Dapirolizumab	Blocking the interaction of CD40-CD40L, B cells, antigen-presenting cells activation	[236]
		CDP7657	Inhibiting CD40L-dependent immune responses	[239]
	Antigen-binding fragment	BI 655,064	Blocking CD40 expressed by B cells and T cells	[238]
Targeting platelet-endothelial cell interaction	PDE3	Cilostazol	Increasing intracellular cAMP levels and inhibiting main protease and Spike glycoprotein; reducing the aggregation of Mpx-expressing neutrophil at the lesion site	[244, 246, 277]
		Dipyridamole	Downregulating various innate immune response genes, such as IL-10, TLR1 and TLR10	[245]
	PDE5	Sildenafil	Regulating proliferation of regulatory T cells, and production of proinflammatory cytokines TNF-α and IL-1β and autoantibodies	[248, 249]
		Vardenafil	Down-regulating M1 macrophage pro-inflammatory markers NOS-2 and TNF-α	[250]
		Tadalafil	Reducing both myeloid-derived suppressor cells and T_reg_ concentrations	[252]
	PAR1	Vorapaxar	Increasing on CD4 and CD8 T cells	[254]
	PAR4	BMS-986,120	Inhibiting thrombin-induced inflammation in astrocytes through Table 2/ERK/NF-κB signaling pathway	[256]
		BMS-986,141		[256]
	Thrombin	Heparin	Regulating T-cells and marginal zone B-cells	[259, 260]
		Dabigatran	Limiting the activation of PAR-1, in turn downregulating sphingosine kinases and disrupting sphingosine-1-phosphate receptor signaling	[261]
	GPIb-V-IX	Anfibatide	Preventing neutrophil NETosis and NET formation and reducing the expression of MAC-1 and P-selectin	[264, 265]
	GPVI	Revacept	Blocking the collagen binding sites	[268]
		Glenzocimab	Blocking the collagen-GPVI pathway	[269]
		Eltrombopag	Inhibiting the GPVI receptor expression and its soluble form	[270]
	GPIIbIIIa	Tirofiban	Causing immune-mediated thrombocytopenia	[272, 273]
		Eptifibatide		[275]
		Abciximab		[275]
		XV459	Blocking platelet activation/aggregation and significantly decrease levels of fibrinogen binding to human platelets	[276]

## Perspectives

Platelets, previously known as the guardians of hemostasis, have gained significant importance in recent years due to exciting discoveries revealing their crucial role in the immune system. Platelets play a vital role in resolving infections and in the development and advancement of immune and inflammatory diseases, either directly or by regulating immune cells. This review uncovers the current evidence for platelet activation in multiple immune-mediated inflammatory diseases and the variety of immune cells that platelets interact with and how these interactions alter their function. First, resting platelets are induced by various factors such as immune complexes through Fc receptors, platelet-targeting autoantibodies and other platelet-activating stimuli, which encourage them to transition from a resting state to an active state in the circulating immune system. Second, activated platelets can release immune activation stimuli, present antigens, and interact with various immune cells. Thirdly, activated platelets regulate the innate immune system, such as neutrophils, monocytes/macrophages, DCs and NK cells, and adaptive immune system, such as T and B cells. Understanding these pathophysiological mechanisms that underlie platelet and immune system interactions may lead to the identification of new therapeutic targets in various immune-mediated inflammatory diseases, which include targeting platelet activation, platelet-immune cell interaction and platelet-endothelial cell interaction. Clinical modulations targeting platelet activation in the immune system include P2Y_12_ receptor inhibitors, COX-1 inhibitors, 12-LOX inhibitors, IVg and SYK inhibitors, TLR7 inhibitors, NLRP3 and BTK inhibitors, IP receptor agonists and AMPK inhibitors. Clinical modulations targeting platelet-immune cell interaction include anti-P-selectin antibody, anti-CD40L antibody and antigen-binding fragment. Clinical modulations targeting platelet-endothelial cell interaction include PDEs inhibitors, FXa inhibitors, PAR1/4 inhibitors, thrombin blockers, GPIb-V-IX inhibitors, GPVI inhibitors and GPIIbIIIa inhibitors.

The comprehensive inhibition of platelet activation in current clinical strategies is reasonable, but the benefit-risk ratio of this strategy is determined by the increased risk of bleeding. Therefore, it is necessary to determine the molecular mechanisms that more specifically target platelet-mediated immune dysfunction. These molecules may inhibit the interactions of platelets and immune cells by blocking adhesion molecules, damaging platelet co-stimulatory molecules, or restricting the release of pro-inflammatory molecules. For example, new molecular mechanisms are constantly emerging in immune-mediated inflammatory diseases. Platelet α-granules are rich in transforming growth factor β1 and PF4, which are associated with the functional reprogramming of myeloid-derived suppressor cells in immune thrombocytopenia [278, 279]. IL-6 blockage, like tocilizumab, alters platelets and the phenotype and function of monocytes in RA, leading to alleviation of several inflammatory and autoimmune conditions [280]. These new molecular mechanisms may become novel clinical treatment methods for immune-mediated inflammatory diseases.

All in all, the interaction mechanism between platelets and immune mechanisms and the successful development of novel drug targets described here, will likely play an integral role in achieving a breakthrough in multiple immune-mediated inflammatory diseases through the direct regulation of platelet activities.

## Data Availability

No datasets were generated or analysed during the current study.

## Abbreviations

ADP: Adenosine diphosphate
5’AMP: 5’adenosine monophosphate
AMPK: AMP-activated protein kinase
APS: Antiphospholipid syndrome
APCs: Antigen-presenting cells
ARDS: Acute respiratory distress syndrome
ATIII: Antithrombin III
ATP: Adenosine triphosphate
BTK: Bruton’s tyrosine kinase
cAMP: Cyclic adenosine monophosphate
CD40L: CD40 ligand
CLL: Chronic lymphocytic leukemia
CXCL: C-X-C motif ligand
CYP450: Cytochrome P450
COX-1: Cyclooxygenase-1
COVID-19: Coronavirus disease 2019
DAMPs: Damage-associated molecular patterns
DCs: Dendritic cells
EBV: Epstein-Barr virus
EPCR: Expressed endothelial protein C receptor
FcαRI: Fcα receptor I
FcεR: Fcε receptor
FcγRIIA: Fcγ receptor IIA
FOXP3: Transcription factor forkhead box protein 3
FVII: Factor VII
FX: Factor X
HCQ: Hydroxychloroquine
HIT: Heparin-induced thrombocytopenia
HMGB1: High mobility group box 1
5-HT: 5-hydroxytryptamine
IBD: Inflammatory bowel diseases
ICAM: Intercellular adhesion molecule
IFN: Interferon
IL: Interleukin
IP: Prostacyclin
IR: Ischemia-reperfusion
ITP: Immune thrombocytopenia
IVIg: Intravenous immunoglobulin
5’GMP: 5’guanosine monophosphate
GP: Glycoprotein
GPCRs: G-protein-coupled receptors
LFA-1: Lymphocyte function-associated antigen-1
12-LOX: 12(S)-lipoxygenase
LPS: Lipopolysaccharide
MAC-1: Macrophage-1 antigen
MHC: Major histocompatibility complex
moDC: Monocyte-derived dendritic cells
mtDNA: Mitochondrial DNA
NETs: Neutrophil extracellular traps
NK: Natural Killer
NF-ĸB: Nuclear factor kappa B
NLRP3: NOD-like receptor family pryrin domain containing 3
NOS: Nitric oxide synthase
NSAIDs: Nonsteroidal anti-inflammatory drugs
PAH: Pulmonary arterial hypertension
PAMPs: Pathogen-associated molecular patterns
PAR: Protease-activated receptor
pDCs: Plasmacytoid dendritic cells
PDEs: Phosphodiesterases
PF4: Platelet factor 4
PGI_2_: Prostaglandin I2
PPH: Primary pulmonary hypertension
RA: Rheumatoid arthritis
ROS: Reactive oxygen species
PSGL1: P-selectin glycoprotein ligand 1
SARS-CoV-2: Severe acute respiratory syndrome coronavirus-2
SDF-1: Stromal cell-derived factor-1
SLE: Systemic lupus erythematosus
SLL: Small lymphocytic lymphoma
SYK: Spleen tyrosine kinase
TF: Tissue factor
TGF-β: Transforming growth factor-β
Th: T helper
TLRs: Toll-like receptors
TNF: Tumor necrosis factor
Tph1: Tryptophan 5-hydroxylase 1
VCAM-1: Vascular cellular adhesion molecule-1
vWF: Von Willebrand factor
